# Hypothesis: The opposing pulling forces exerted by spindle microtubules can cause sliding of chromatin layers and facilitate sister chromatid resolution

**DOI:** 10.3389/fgene.2023.1321260

**Published:** 2023-11-24

**Authors:** Joan-Ramon Daban

**Affiliations:** Department of Biochemistry and Molecular Biology, Autonomous University of Barcelona, Barcelona, Spain

**Keywords:** chromosome structure, mitotic chromosome, multilayer chromatin, sister chromatid resolution, mitosis, multilayer chromosome

## Abstract

Previous studies indicated that mitotic chromosome structure consists of many stacked layers formed by a mononucleosome sheet folded as a helicoid. This multilayer chromatin structure justifies the cylindrical shape of chromosomes and the transverse orientation of cytogenetic bands, and can explain chromosome duplication by the formation of a transient double helicoid that is split into two sister chromatids in mitosis. Here it is hypothesized that the bipolar pulling forces exerted by the mitotic spindle cause the sliding of the layers and facilitate sister chromatid resolution. This hypothesis is supported by three favorable conditions: i) There is no topological entanglement of DNA between adjacent layers; ii) The orientation (parallel to the stacked layers) of the bipolar kinetochore microtubules is adequate to produce layer sliding in opposite directions; iii) The viscous resistance to the sliding caused by the weak interactions between nucleosomes in adjacent layers can be overcome by the microtubule pulling forces.

## 1 Introduction

Genomic DNA of eukaryotes is divided into large fragments that are packed within chromosomes. Growing cells replicate their DNA in interphase and the resulting two sets of chromosomes are condensed and precisely distributed into the daughter cells in mitosis (Sumner, 2003). This part of the cell cycle is very complex and requires the separation of the two chains of duplicated DNA, which are resolved into two sister chromatids without causing any damage to the original DNA sequence. Chromatids attached to microtubules are pulled to the opposite poles of the mitotic spindle (Petry, 2016) and then cytokinesis yields two daughter cells.

Throughout the cell cycle, chromosomal DNA molecules are associated with histone proteins and form long chromatin filaments filled with many nucleosomes. Each nucleosome is composed of a flat cylindrical core particle (11 nm diameter and 5.7 nm height) formed by ∼146 bp of DNA wrapped around an octamer of core histones (Luger et al., 1997). In the chromatin filaments, nucleosome cores are connected with short segments of linker DNA that are associated with histone H1 (Fierz and Poirier, 2019). Several structural models have been proposed for the condensation of the chromatin filament into micrometer-sized mitotic chromosomes [reviewed in (Piskadlo and Oliveira, 2016; Fierz, 2019; Beseda et al., 2020)]. It was proposed that the chromatin filament is highly disordered in the chromosomes (Eltsov et al., 2008; Nishino et al., 2012), but generally it is considered that the filament forms radial loops. Early studies suggested that the loops are attached to a central non-histone protein scaffold (Paulson and Laemmli, 1977). However, chromosome stretching experiments indicated that proteins do not form a continuous backbone within chromosomes (Poirier and Marko, 2002) and it was suggested that their mechanical integrity is due to a chromatin network crosslinked by non-histone proteins. A more recent version of the radial-loop model is based on results obtained using genome-wide chromosome conformation capture (Hi-C) techniques combined with polymer simulations of chromatin fibers (Lieberman-Aiden et al., 2009), which led to the proposal that mitotic chromatin forms nested loops of ∼0.5 Mb (consisting of ∼400-kb outer loops and ∼80-kb inner loops) mediated by condensin (Gibcus et al., 2018). Furthermore, according to early microscopic observations (Ohnuki, 1965; Rattner and Lin, 1985; Boy de la Tour and Laemmli, 1988; Câmara et al., 2023), these authors suggested that the loops are organized forming a helical array. Other recent reports based on Hi-C analysis of different species (Schloissnig et al., 2021; Kuvalová et al., 2023) also consider that chromatin in chromosomes is helically folded.

**FIGURE 1:**
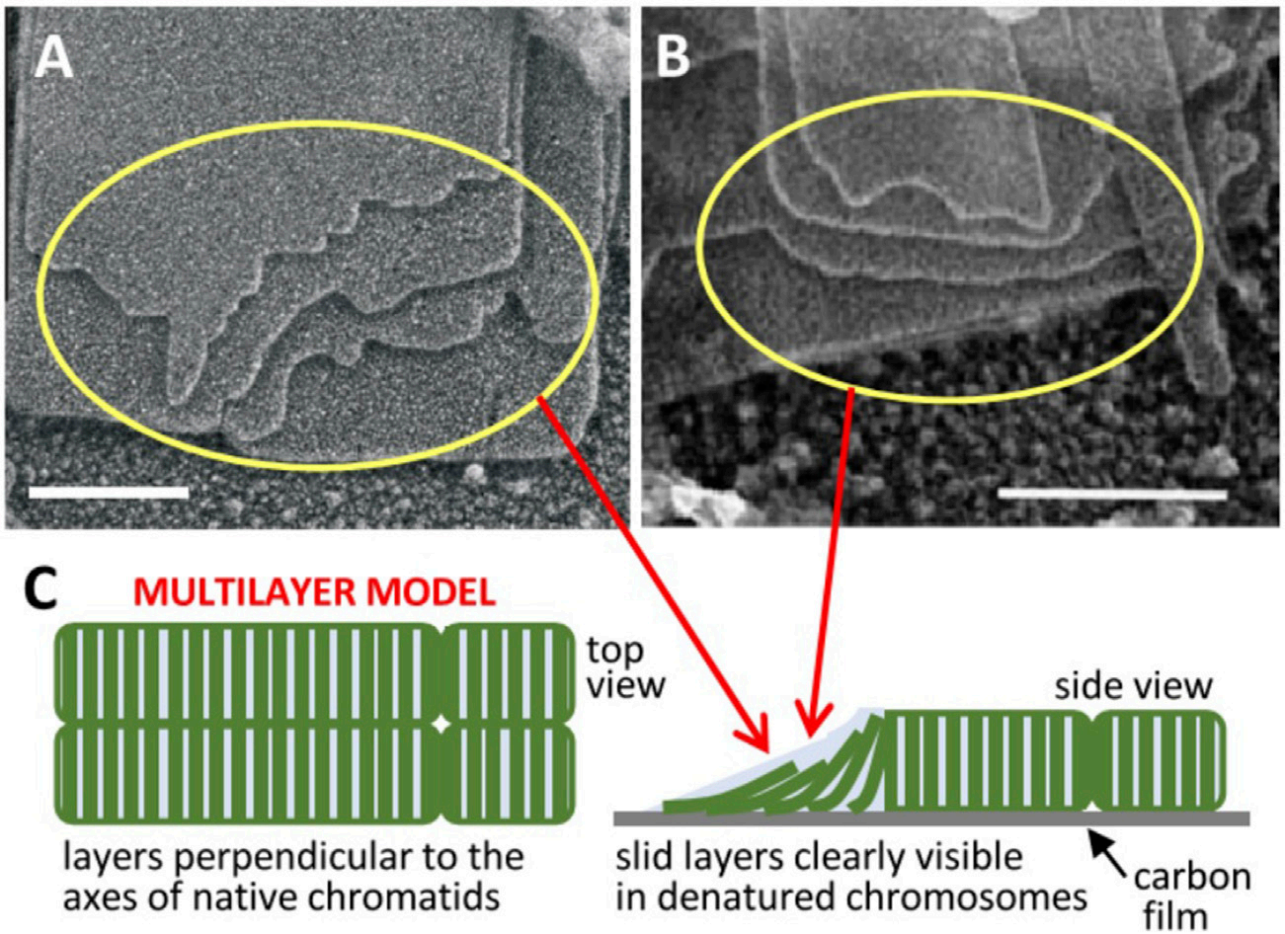
Multilayer structure of mitotic chromosomes. Transmission electron microscopy images of multilaminar plates emanated from human **(A)** (Castro-Hartmann et al., 2010) and chicken **(B)** (Gállego et al., 2009) chromosomes under metaphase ionic conditions (scale bars 200 nm); the sliding of successive layers can be clearly seen in regions indicated by yellow rings. **(C)** The observed layer sliding (schematized in the unfolded telomere region pointed by red arrows) and other results reviewed in the text suggested a stacked multilayer structure of chromatin in native metaphase chromosomes. Figures reproduced with permission from references (Castro-Hartmann et al., 2010) **(A)**, (Gállego et al., 2009) **(B)**, and (Daban, 2021a) **(C)**.

It was observed that incubation of human and chicken chromosomes at 37°C [under metaphase ionic conditions including Mg^2+^ (Strick et al., 2001)] on electron microscopy grids caused the emanation multilayered plates (Caravaca et al., 2005; Gállego et al., 2009). In buffers containing the divalent cation chelator EDTA, these planar structures are unfolded and the chromatin filaments become visible (Gállego et al., 2009) but, in aqueous solutions containing Mg^2+^, atomic force microscopy experiments showed that the chromatin filament forms a mechanically resistant planar network, which is stable at room temperature (Gállego et al., 2010). It was proposed that chromatin in metaphase is folded into many stacked thin plates oriented perpendicular to the chromosome axis [Figure 1 (Gállego et al., 2009; Castro-Hartmann et al., 2010)]. This chromosome organization was unexpected (Daban, 2011), but early work showed that in dinoflagellate chromosomes, which do not contain histones, DNA is packed forming a multilayer liquid-crystal structure (Livolant and Bouligand, 1978; Rill et al., 1989; Mitov, 2017). In agreement with electron microscopy and atomic force microscopy results, cryo-electron tomography studies showed that frozen-hydrated chromatin (not adsorbed to any flat substrate) emanated from human metaphase chromosomes is planar and forms multilayered plates (Chicano et al., 2019). The tomographic three-dimensional reconstructions showed that in the plates each layer has a thickness of ∼6 nm, corresponding to a sheet of slightly tilted nucleosomes. Furthermore, X-ray scattering of whole chromosomes under metaphase ionic conditions showed a dominant peak at ∼6 nm that can be correlated with the repetitive distance between stacked layers, in which the nucleosomes of adjacent layers are interacting thought their lateral faces (Chicano et al., 2019). Furthermore, cryo-tomograms showed large multilayer plates with widths similar to the diameter of human metaphase chromatids. Consistent with results indicating that chromosomes are helical structures (see above), it was proposed that the successive chromatin layers are connected forming a continuous helicoid (Daban, 2014) containing ∼0.5 Mb per turn in human chromosomes. Since the long nested loops considered in Hi-C experiments presented in the preceding paragraph must be highly packed to achieve the high nucleosome density of metaphase chromosomes (Daban, 2003; Eltsov et al., 2008; Ou et al., 2017; Chen et al., 2023), it was suggested that they could be compacted into chromatin layers (Chicano et al., 2019). Other authors (Grigoryev et al., 2016; Bascom and Schlick, 2017) suggested a hierarchical layering of loops to integrate the models based on a highly disordered chromatin filaments (Eltsov et al., 2008; Nishino et al., 2012) and results indicating a multilayer organization of chromatin in chromosomes. Electron diffraction analysis revealed a repetitive structure (100–200 nm) oriented perpendicular to the chromosome axis (Hayashida et al., 2021), which could correspond to clusters of stacked chromatin layers.

## 2 Dynamic properties, relationship with cytogenetic observations, and possible functional roles of multilayered chromosomes

Metaphase chromosomes of different plant and animal species are elongated cylinders having relatively similar shape proportions (Daban, 2014) and about the same DNA density (∼166 Mb/μm^3^) (Daban, 2000). This indicates that presumably chromatin is condensed in chromosomes according to a general structural pattern. Furthermore, chromatin fragments obtained by micrococcal nuclease digestion of human metaphase chromosomes associate spontaneously, under metaphase ionic conditions, forming multilayered plates having the same structural characteristics as plates emanated from native chromosomes (Milla and Daban, 2012). These findings suggested that chromosomes could be self-organized supramolecular structures (Daban, 2014) such as other micrometric multilayer structures formed by the spontaneous assembly of different repetitive subunits of biological origin, including viruses (Adams et al., 1998), purified nucleosome core particles (Leforestier et al., 2001; Mangenot et al., 2003), and DNA (Wagenbauer et al., 2017). Chromatin has the typical properties observed for different soft-matter systems (Daban, 2021b), in which the weak interactions between repetitive building blocks (with an interaction energy comparable to the background thermal energy) lead to spontaneous pattern formation (Jones, 2002; Ross, 2016; Van der Gucht, 2018). It can be considered that chromosomes have a lamellar liquid-crystal order, but at the same time they are hydrogels because their building blocks (nucleosomes) are chemically crosslinked by linker DNA.

It has been shown that the different internucleosome interaction energies in different regions of this multilayer structure provide a consistent physical explanation of the elongated smooth cylindrical shape of metaphase chromosomes and of their mechanical properties (Daban, 2014). The nucleosomes within each layer are strongly associated by the covalent backbone of linker DNA, but there is only a weak electrostatic face-to-face interaction between nucleosomes in adjacent layers. This weak interlayer association is consistent with the observation that many plates emanated from chromosomes show a relative sliding between the successive layers [Figures 1A, B (Gállego et al., 2009; Castro-Hartmann et al., 2010)]. It is also consistent with the easy deformation and elastic properties of chromosomes observed in stretching experiments performed with micropipettes (Poirier et al., 2000). Nucleosomes in the periphery of the chromosome are in contact with the medium; they cannot fully interact with bulk chromatin within layers and this generates a destabilizing surface energy. Chromosomes are smooth cylinders because this morphology has a lower surface energy than structures having irregular surfaces. Furthermore, nucleosomes in the telomere surface can interact only with the nucleosomes of one layer. In contrast, nucleosomes in the chromosome lateral surface are less exposed to the medium than those in the telomere because they interact with the nucleosomes of two adjacent layers. These energy differences cause a symmetry breaking and justify a minimum energy and consequently maximum stability for the elongated shape of chromosomes (Daban, 2014).

The multilayer organization of chromosomes provides a structural framework for interpreting cytogenetic results that cannot be justified by other structural models (Daban, 2015). It was proposed that the typical bands of human chromosomes (Sumner, 2003; Chai and Li, 2023) are produced by the preferential staining of clusters of chromatin layers with different dyes, and that the observed transverse orientation of the bands is due to the perpendicular orientation of the chromatin layers with respect to the chromosome axis. The weak interlayer interactions (see the preceding paragraph) explain the splitting of broad bands (formed by several layers) into thinner sub-bands observed in chromosome stretching experiments (Hliscs et al., 1997). Cytogenomic results showed that there are very thin bands containing ∼1 Mb (International Human Genome Sequencing Consortium, 2001; Daban, 2021a; Liehr, 2021), indicating that relatively short sequences of DNA can fill completely the cross-section of the chromosome, and this is also compatible with a multilayered organization of chromosomes in which each layer is built with ∼0.5 Mb. Moreover, this chromosome structure explains the orthogonal orientation and planar structure of the connection surfaces observed in sister chromatid exchanges and in cancer chromosome translocations (Daban, 2015; Daban, 2021a).

In buffers containing interphase cation concentrations (Strick et al., 2001), the chromatin emanated from G1, S, and G2 nuclei also has a planar morphology (Chicano and Daban, 2019). Furthermore, chromatin fragments obtained from nuclei (digested with micrococcal nuclease) in G1, S, and G2 cell cycle phases associate to form plate-like structures. In agreement with these observations, cytogenetic experiments using microdissection-based multicolor banding showed that the chromosome band pattern is maintained during all the stages of the interphase (Lemke et al., 2002; Weise et al., 2002; Yurov et al., 2007), suggesting that the chromosome territories observed in interphase (Cremer and Cremer, 2010) retain the layered structure of metaphase chromosomes. However, the plates observed in interphase have a low tendency to form the multilayered structures observed in mitotic chromosomes, suggesting that they are not so tightly stacked (Chicano and Daban, 2019). This causes a higher exposure to the medium that could facilitate gene expression and DNA replication. This structural change has been interpreted as a chromosome phase transition (Daban, 2021b), which may be related to the sudden disappearance of the condensed morphology of chromosomes observed when the cell enters interphase (Skeen et al., 1993).

According to Hi-C experiments in interphase, chromatin is organized into topologically associating domains (TADs), which are considered to be the functional subunits of chromatin, and larger compartments (Lieberman-Aiden et al., 2009; Bonev and Cavalli, 2016). The loops of the chromatin filament proposed from the results obtained in Hi-C studies can be interpreted as contacts produced by the folding of the filament within layers. Since the size of TADs (0.2–1 Mb) is similar to the amount of DNA in a chromatin layer, it was proposed that each layer may correspond to a TAD (Chicano and Daban, 2019), and that presumably the functional insulation between layers is produced by the proteins CTCF (Nora et al., 2017) and cohesin (Rao et al., 2017; Liu and Dekker, 2022). During mitosis, the most frequent contacts observed in Hi-C experiments involve very distant sequences (Naumova et al., 2013; Nagano et al., 2017) and this could be due to a stacking of chromatin layers that favor contacts between several successive layers (Daban, 2020). The tight stacking of chromatin layers in mitosis may be responsible of the observed inhibition of transcription. In contrast, it is expected that chromatin accessibility increases greatly when the layers become unstacked in euchromatin compartments during interphase and DNA becomes available for the interaction with transcription factors, activators, and mediator (Chen et al., 2022) from the two sides of the exposed chromatin layers. Considering that there is a switching between active and inactive compartments during cell differentiation (Zheng and Xie, 2019), it was suggested that only specific clusters of layers are fully unstacked and active in different cell types (Daban, 2021b). Various epigenetic elements [DNA methylation, histone variants and post-translational modifications, HP1 and polycomb proteins, non-coding RNAs (Luger et al., 2012; Cortini et al., 2016; Cavalli and Heard, 2019)] could modulate the accessibility of chromatin layers in the distinct stages of cell differentiation.

## 3 Chromosome duplication: the bipolar spindle pulling forces can cause sliding of chromatin layers that may contribute to sister chromatid resolution

During the S period of interphase, nucleosomes are temporarily dissociated in many replication origins to allow the interaction of DNA with all the replisome components and eventually two daughter chromatin filaments are produced (Alabert and Groth, 2012). As indicated in the Introduction, the observation of planar chromatin emanated from chromosomes and previous results showing that chromosomes have a helical morphology suggests that the nucleosome filament within chromosomes forms a helicoid. The successive turns of this helicoid have a thickness corresponding to a mononucleosome sheet that must be disrupted to allow DNA synthesis. Presumably, the replicated DNA associated with histones also forms planar chromatin and the complete duplication of the chromosome originates a transient double helicoid (Daban, 2020). This structure has topological properties that may facilitate DNA repair by homologous recombination, which is known to occur during lateS-G2 phase (Misteli and Soutoglou, 2009). Duplicated chromosomes organized as double helicoids contain two copies of all the sequences of the genome in close proximity between them, and if a double-stranded DNA break is produced in any sequence the other copy can be used as a homologous template for the repair (Daban, 2020).

**FIGURE 2:**
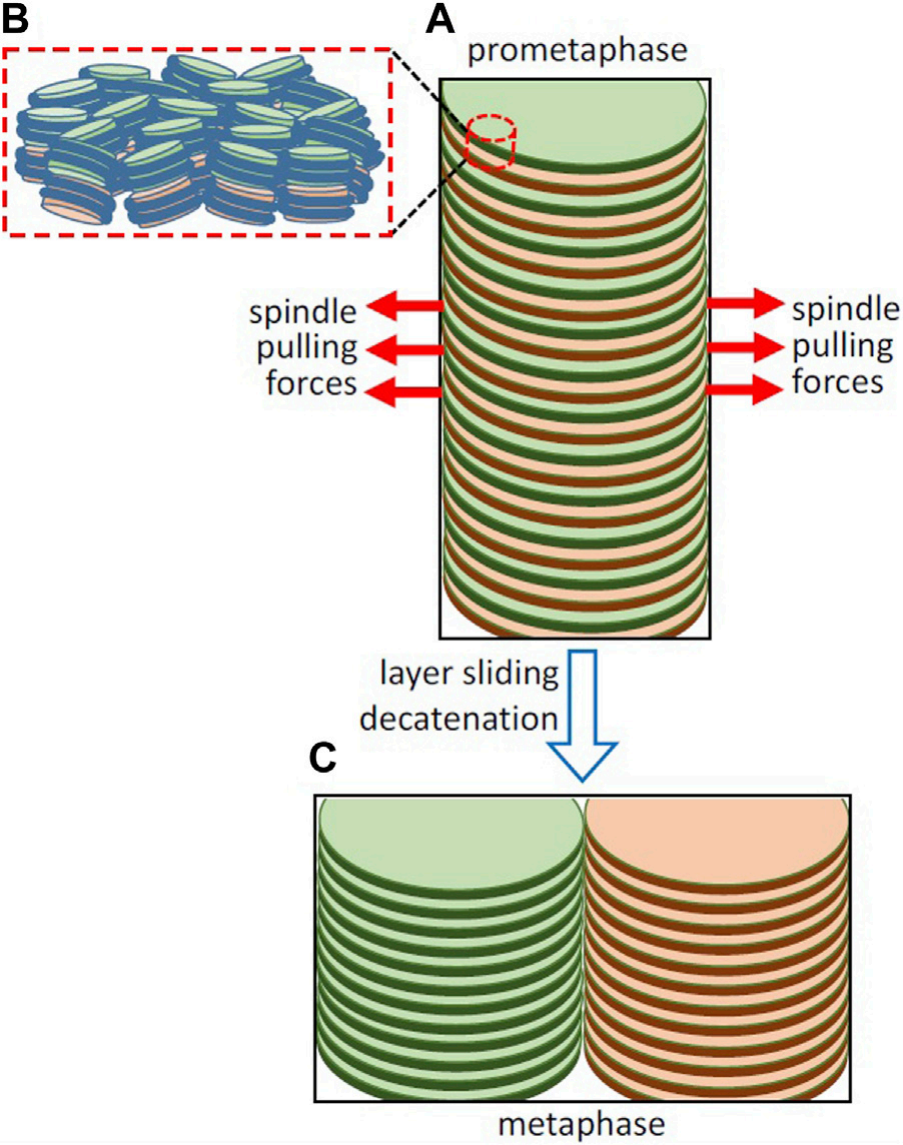
Hypothetical involvement of the opposite pulling forces exerted by the spindle in layer sliding and sister chromatid resolution. **(A)** Simplified representation of part of a replicated chromosome forming a double helicoid and of the spindle pulling forces in early prometaphase. The organization of nucleosomes in two adjacent layers is schematized in **(B)**; the path of DNA joining the nucleosomes in each layer is not known at present and it is not included in the figure. **(C)** Layer sliding caused by the bipolar spindle forces, decatenation by topoisomerase II, and the energetically favorable stacking of chromatin layers leads to the complete sister chromatid resolution in metaphase. Chromosomes in living cells are not so perfectly regular as in the idealized representations in this figure, as they are soft condensed matter structures that are subject to local thermal fluctuations and are easily deformable by external forces (Daban, 2021b).

Scanning electron microscopy results showed that during early-prophase individual chromosomes are not distinguishable; at later stages of prophase chromosomes are long cylindrical structures, usually continuous, but sometimes they are segmented into blocks (Sumner, 1991). The morphology of these chromosomes and their circular cross-section (with a diameter of ∼1.3 µm in the case of human chromosome 2) indicates that they are clearly not split into separate chromatids at this stage of mitosis. Metaphase chromosomes are split into two cylindrical chromatids (each one with a diameter of ∼1.0 µm in the case of human chromosome 2), which are about half the length of the prophase chromosomes (Sumner, 1991). In agreement with these observations, results obtained applying three-dimensional fluorescence deconvolution microscopy over time to diverse mammalian cells (Liang et al., 2015) showed an increase of chromosome width with no change in chromosome length in late prophase, and a further increase in width and a dramatic decrease in length during the prometaphase-to-metaphase stage. The model described above in which it is proposed that replicated chromosomes form a double helicoid is compatible with these results. According to this model (see the simplified scheme in Figure 2), it is expected that each one of the split chromatids should have approximately one-half of the helicoidal turns of the prophase chromosome, and consequently their length should be reduced to one-half. Furthermore, as observed experimentally, the expected total width of the metaphase chromosome split into two helicoidal chromatids should be roughly two times the diameter of the double helicoid in prophase.

The structure and dynamic properties of the mitotic spindle are based on the self-organization of microtubules and motor proteins (Oriola et al., 2018). In animal cells, centrosomes promote spindle bipolarization after the breakdown of the nuclear envelope. Plant cells do not possess centrosomes and the initial bipolarization of the spindle microtubules occurs on the nuclear envelope (Liu and Lee, 2022). During prometaphase the pushing and pulling forces of microtubules and motor proteins cause the congression of chromosomes to the spindle equator (Maiato et al., 2017). Topoisomerase II and condensin are located along the single axis of the mid-prophase chromosome and then they are associated with the axes of the split chromatids during prometaphase (Liang et al., 2015). Most cohesin is dissociated from the prophase chromosomes, but the remaining cohesin holds the two sister chromatids together up to the onset of anaphase (Shintomi and Hirano, 2010). The connection of the two chromatids is released by separase-mediated cleavage of cohesin (Uhlmann et al., 1999; Hauf et al., 2001) and the daughter chromosomes are segregated to the opposite poles at the end of anaphase.

Kinetochores are tightly bound to centromeres (Yatskevich et al., 2022). The forces generated by the opposing microtubules attached to kinetochores produce tension between sister chromatids (Waters et al., 1996; Hara and Fukagawa, 2020). In grasshopper spermatocytes, the pulling forces exerted by microtubules during prometaphase (∼0.1 nN) are larger than those observed during anaphase (Nicklas, 1988). This is surprising because the highest microtubule forces are expected to be required to produce the complete separation of sister chromatids during anaphase. Taken together, these observations suggest that the relatively high pulling forces observed in prometaphase could be involved in the resolution of sister chromatids. Here it is hypothesized that the opposite pulling forces exerted the bipolar spindle may produce sliding of the chromatin layers in the double helicoid (Figure 2A). The pulling forces are oriented parallel to the chromatin layers and can be used to overcome the viscous resistance to the sliding on opposite directions of alternate layers belonging to the two sister chromatids. In this multilaminar structure, the viscosity is generated by the weak face-to-face interactions between nucleosomes of adjacent layers (Figure 2B). On the other hand, nanotribology experiments indicated that chromatin layers are mechanically resistant to high stretching forces (Gállego et al., 2010); scanning with atomic force microscopy tips applying lateral forces up to ∼5 nN did not cause any irreversible alteration of mononuclesome layers. Therefore, it is expected that the spindle pulling forces can be applied without causing permanent deformation of planar chromatin in the layers. This sliding is produced in the centromere region and presumably generates separation of chromatids only in a limited part of the whole structure. Nevertheless, this initial sliding caused by the opposite pulling forces gives rise to an unstable structure that could spontaneously evolve into a more stable condensed structure (Daban, 2014) consisting of two completely resolved chromatids having a minimum energy (Figure 2C).

Note that the proposed sliding leading to chromatid resolution is not possible if DNA crosslinks the adjacent layers of the two helicoids. The easy sliding of layers in chromatin plates (see above), which was inferred from the frequently observed displacement of the edges of successive layers in chromatin plates emanated from metaphase chromosomes [Figures 1A, B (Gállego et al., 2009; Castro-Hartmann et al., 2010)], indicates that there is no entanglement of DNA between consecutive helicoidal turns. In contrast to the enormous topological complexity expected for extended chromatin fibers, the simple and well-defined topological organization of multilayer chromosomes may greatly facilitate chromosome duplication. However, the resulting double helicoid has intrinsic topological links that must be considered. In the case of the double helix of DNA (which is used here as a reference) each turn of the helix produces a topological bond between the two DNA chains that can only be removed if the covalent backbone of one of the two chains is transiently cut (Bates and Maxwell, 1993). In replicated multilayer chromosomes, there are two double-stranded DNA molecules that form two helicoids that fill progressively the chromosome from one telomere to the other (Daban, 2021a), and consequently each turn generates a topological link. Since this entanglement is produced between two double-stranded DNA molecules, the two chains of one of the two DNA molecules must be cut to allow the passage of the other double helix to remove a single topological bond. This problem can be effectively resolved by the cell. Early results (Giménez-Abián et al., 1995) demonstrated that topoisomerase II, which can pass one DNA segment through another via a transient double-strand break (Berger et al., 1996), is located in the axis of prophase chromosomes (see above) and is necessary for the decatenation of sister chromatids during prometaphase. More recent results (Piskadlo et al., 2017) indicated that the interplay between condensin and topoisomerase II is required for the complete decatenation of sister chromatids.

## 4 Discussion

The proposed mechanism for sister chromatid resolution is based on the sliding in opposite directions of the alternating chromatin layers of the double helicoid produced after chromosomal DNA replication. Further removal of topological links by topoisomerase II (see above) and the spontaneous stacking of the chromatin layers (Daban, 2014) generates two completely condensed and stable minimum-energy chromatids. Presumably this mechanism is not altered if double-stranded DNA breaks are repaired by homologous recombination [see Section 3 (Daban, 2020)] before layer sliding. Mitotic homologous recombination between sister chromatids maintains genomic stability because it allows the precise repair of double-strand breaks but, when the recombination repair occurs between homologous chromosomes, it can produce loss of heterozygosity, and the repair by recombination between heterologous chromosomes can produce reciprocal translocations (Moynahan and Jasin, 2010). It is reasonable to speculate that, after these repair reactions, layer sliding in opposite directions and spontaneous layer stacking will facilitate the resolution of the resulting recombinant sister chromatids. In meiotic cells (Ur and Corbett, 2021), this mechanism based on bipolar spindle forces and the formation of final minimum-energy structures could be involved in the segregation of the recombined homologous chromosomes in meiosis I, and in the resolution of the resulting sister chromatids in meiosis II.

It was suggested that sister chromatids have opposite handedness (Boy de la Tour and Laemmli, 1988), but it was argued (Daban, 2020) that such a mirror symmetry is very unlikely because it would require the existence of two different sets of transcription machineries and specific factors sterically compatible, respectively, with the structure of the left- and right-handed helicoidal chromatids. Furthermore, concerning the structural feasibility of the proposed chromosome replication intermediates, it must be taken into account that it is geometrically impossible to construct a double helicoid from two helicoids with opposite handedness. Therefore, it seems reasonable to consider that all chromosomes have the same handedness. In fact, single-handed (homochiral) forms are very common in living systems. Homochirality is observed in basic building blocks (such as amino acids and nucleotides), in the structure of their polymers (proteins and nucleic acids), and in the resulting higher-order structures (Barron, 2009). In particular, the typical B-form DNA double helix is right-handed and the DNA in nucleosomes forms a left-handed superhelix (Richmond and Davey, 2003). Since it is known that different chemical and biological chiral building blocks can induce the formation of chiral liquid crystals (Livolant and Leforestier, 2000; Hunyadi et al., 2007; Gibaud et al., 2012; Zhang et al., 2016), it could be that the handedness of chromosomes is related to the homochirality of nucleosomes. In favor of this possibility, *in vitro* studies performed with purified nucleosome cores showed that the chiral nature of these particles induce the formation of large liquid crystalline columnar phases having a left-handed helical shape (Livolant and Leforestier, 2000). Unfortunately, the handedness of chromosomes is not known at present. Note however that the hypothesized sliding of chromatin layers is structurally compatible with either right- or left-handed chromosomes.

## Data Availability

The original contributions presented in the study are included in the article, further inquiries can be directed to the corresponding author.
